# Hand-held cow horn: resurgence of an old arm or apotential terrorist weapon

**DOI:** 10.5249/jivr.v4i1.104

**Published:** 2012-01

**Authors:** Lawal Khalid, Adamu Ahmed

**Affiliations:** ^*a*^Division of General Surgery, Department of Surgery, Ahmadu Bello University Teaching Hospital, Zaria, Nigeria.

## Abstract

A 23 year old man presented with intestinal evisceration from stab injury to the left side of the abdomen with a hand-held cow horn at a local night party. He complained of severe abdominal pain and bleeding at the site of injury. He was hemodynamically stable. At emergency exploration, the eviscerated bowel was viable with no adjacent mesenteric tear. Other intra abdominal organs were normal. The eviscerated bowel was lavaged and reduced into the abdomen through the 7cm anterior abdominal wall laceration. The laceration was repaired and abdomen closed in layers. Post operative recovery was uneventful. The hand-held cow horn can easily be concealed and may pass through security checks undetected. It should be added to the ever increasing list of weapons of small scale terror.

## Introduction

**Figure 1: A detached and decorated cow horn.**
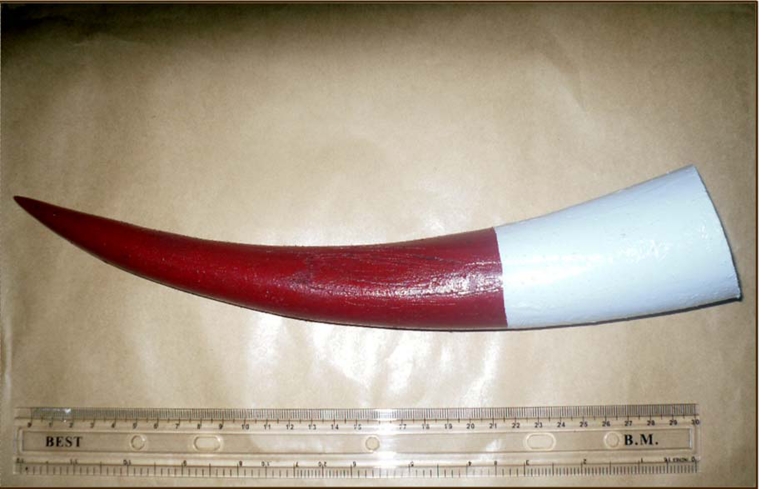


Detached cow horns are used widely in some cultures for internal decorations (Figure.1). Unfortunately the same horn can be used for other detrimental purposes including assault. The detached organ is easily trimmed to size for handling and the pointed end could inflict injury. Although there are several reports on cow gore injury,^1,3,4^injury from hand-held cow horn is rarely reported. In this world of ever increasing terrorist's activities, this surely is a potential weapon of small scale terror. This is a report of serious morbidity caused by this ‘new weapon’.

## Case Report

A 23 year old young Fulani man presented to the Accident & Emergency room in the early hours of the morning with history of stab in the abdomen with a hand-held cow horn. An hour earlier he was at a local party with his peers when a scuffle ensued and he was stabbed with a cow horn on the left side of the abdomen. He had severe abdominal pain and noticed that his intestine was protruding through the wound. He was helped to the hospital by his friends. There was no loss of consciousness. He bled from the site of injury. No vomiting or abdominal distension. There was no hematuria. Examination revealed an anxious young male who was not pale. He did not have any alcohol smell or evidence of any parenteral drug abuse, but no objective alcohol test was done. There was tachycardia of 92/min and a blood pressure of 100/60mmHg. Chest examination was normal. There was an obvious eviscerated hyperemic loop of bowel in the left paraumbilical region. The abdomen was moderately tender but there was no evidence of significant free peritoneal fluid collection. Rectal exam was normal. 

He was resuscitated with intravenous fluids and antibiotics, and tetanus prophylaxis was given. Eviscerated bowel was covered with sterile warm moist gauze soaked in saline. Complete blood counts, electrolytes and urea laboratory test results were within normal limits. Abdominal ultrasonography suggested minimal fluid collection in the peritoneal cavity. At surgery, the eviscerated bowel was found to be viable. It was thoroughly lavaged with saline. Via a midline incision, the abdomen was then opened. The peritoneal cavity was found to be relatively clean with just about 30 mls of hemoperitoneum in the pelvis. There was a 7 cm transverse rent involving all layers of the anterior abdominal wall to the left of the umbilicus, through which about 35 cm loop of jejunum eviscerated. There was no mesenteric injury. All other abdominal organs were normal. The eviscerated bowel was reduced with ease. The laceration was debrided and repaired in layers. The abdomen was lavaged with normal saline and the wound closed in layers. Post operative recovery was uneventful and he was discharged home on the 8th post operative day. The patient has remained well and stable at 12-month follow-up.

## Discussion

This report aims at highlighting how a seemingly innocuous hand-held cow horn could cause serious injury and instill fear and scare amongst innocent people.

Abundant literature exists on cow gore causing variable degrees of injury. The abdomen is the commonest region so affected.^1-3^A majorities of these injuries occurs amongst cow rearing populations, although a few incidences are recorded during bull plays. The cow’s main defense is the use of its horn to deter potential threat or chase away invading enemy. However, when the organ (horn) is detached, it is of not much use to man except for in-house decorations as practiced by certain communities. Unfortunately it may have some other harmful use as in this report. The detached organ is cut and trimmed to size towards its pointed end, and is easily pocketed or hidden under the garment. It is brought out when the need arises and the situation permits. An otherwise enjoyable party came to an abrupt end as the outcry from the stabbed victim and the evisceration of bowel caused scare and pandemonium.

The site of stab could potentially injure vital organs- bowel and mesentery; (L) kidney and ureter; tail of pancreas and any of the great abdominal vessels on the left. Injury to any of these would have been more catastrophic. Luckily in our patient the injury was restricted to the anterior abdominal wall muscles. We therefore had to deal with the problem of eviscerated bowel and the potential inoculums of microbes associated with the horn. As the patient was brought to hospital early, contamination and desiccation of the bowel was minimal and features of obstruction and strangulation had not supervened. In addition, broad spectrum intravenous antibiotics including metronidazole were administered prophylactically. Thus, prompt reporting and resuscitation, absence of significant intra abdominal injury, as well as early surgical intervention are thought to have contributed to the successful outcome in our patient.

This type and cause of injury adds a new dimension of violence and small scale terror. The magnitude of terror could be amplified if the assault is directed at the pilot of an aircraft, captain of a ship, or driver of a vehicle, as it is enough to change the direction and destination of a large number of people! Furthermore, this modified weapon consists of keratin and some structured proteins like tubulin and elastin. It does not contain any metal and may therefore not ignite the alarm of metal detector. Although we have not been able to see how it would look like on airport scanner, we believe that the pointed end could easily be hidden by interposition with similar non pointed piece of horn. The detached cow horn, is handy, may be easily concealed and could be a weapon of small scale terror. It could also be well decorated as a camouflage to divert attention from its potential terrorist’s unsuspecting weapon. It should be added to the ever increasing list of weapons of violence and terror.
